# Identification of Insulin-like Growth Factor (IGF) Family Genes in the Golden Pompano, *Trachinotus ovatus*: Molecular Cloning, Characterization and Gene Expression

**DOI:** 10.3390/ijms25052499

**Published:** 2024-02-21

**Authors:** Charles Brighton Ndandala, Qi Zhou, Zhiyuan Li, Yuwen Guo, Guangli Li, Huapu Chen

**Affiliations:** 1 Guangdong Provincial Key Laboratory of Pathogenic Biology and Epidemiology for Aquatic Economic Animals, Guangdong Research Center on Reproductive Control and Breeding Technology of Indigenous Valuable Fish Species, Fisheries College, Guangdong Ocean University, Zhanjiang 524088, China; charlesndandala@gmail.com (C.B.N.); xiaopangzhou163@163.com (Q.Z.); g_yl903@163.com (Z.L.); gdougyw@163.com (Y.G.); guangligdou@163.com (G.L.); 2 Southern Marine Science and Engineering Guangdong Laboratory (Zhanjiang), Zhanjiang 524025, China

**Keywords:** IGFs, cloning, fasting, sex steroids, fish reproduction, *Trachinotus ovatus*

## Abstract

Insulin-like growth factors (IGFs) are hormones that primarily stimulate and regulate animal physiological processes. In this study, we cloned and identified the open reading frame (ORF) cDNA sequences of IGF family genes: the insulin-like growth factor 1 (IGF1), insulin-like growth factor 2 (IGF2), and insulin-like growth factor 3 (IGF3). We found that IGF1, IGF2, and IGF3 have a total length of 558, 648, and 585 base pairs (bp), which encoded a predicted protein with 185, 215, and 194 amino acids (aa), respectively. Multiple sequences and phylogenetic tree analysis showed that the mature golden pompano IGFs had been conserved and showed high similarities with other teleosts. The tissue distribution experiment showed that IGF1 and IGF2 mRNA levels were highly expressed in the liver of female and male fish. In contrast, IGF3 was highly expressed in the gonads and livers of male and female fish, suggesting a high influence on fish reproduction. The effect of fasting showed that IGF1 and mRNA expression had no significant difference in the liver but significantly decreased after long-term (7 days) fasting in the muscles and started to recover after refeeding. IGF2 mRNA expression showed no significant difference in the liver but had a significant difference in muscles for short-term (2 days) and long-term fasting, which started to recover after refeeding, suggesting muscles are more susceptible to both short-term and long-term fasting. In vitro incubation of 17β-estradiol (E_2_) was observed to decrease the IGF1 and IGF3 mRNA expression level in a dose- (0.1, 1, and 10 μM) and time- (3, 6, and 12 h) dependent manner. In addition, E_2_ had no effect on IGF2 mRNA expression levels in a time- and dose-dependent manner. The effect of 17α-methyltestosterone (MT) in vitro incubation was observed to significantly increase the IGF3 mRNA expression level in a time- and dose-dependent manner. MT had no effect on IGF2 mRNA but was observed to decrease the IGF1 mRNA expression in the liver. Taken together, these data indicate that E_2_ and MT may either increase or decrease IGF expression in fish; this study provides basic knowledge and understanding of the expression and regulation of IGF family genes in relation to the nutritional status, somatic growth, and reproductive endocrinology of golden pompano for aquaculture development.

## 1. Introduction

The golden pompano, *Trachinotus ovatus*, is an economically important saltwater fish in China, belonging to the Carangidae family [1,2]. Due to its fast growth and adaptability for cage culture, it is a suitable fish for aquaculture [2]. The annual production of golden pompano has reached 240,000 tons [2]. *T. ovatus* is a highly sought-after fish species due to its mild, sweet flavor and firm, white flesh. It is also known for its high nutritional value, being an excellent source of omega-3 fatty acids, vitamins, and minerals. It is an excellent source of essential nutrients and can help to improve overall health and reduce the risk of certain diseases [2]. For these reasons, it is becoming increasingly popular as a dietary supplement and a source of nutrition for those looking to improve their health and well-being [2]. In recent years, the golden pompano fish has become the subject of considerable research due to its potential health benefits, particularly regarding insulin-like growth factors (IGFs). IGFs are peptides mainly produced in the liver and other tissues. IGFs plays an important role in fish growth, reproduction, and other physiological processes, including the regulation of cell proliferation, differentiation, and metabolism [3,4,5,6]. IGFs have been identified in numerous fish, including spotted scat, seabream, salmonids, zebrafish, tilapia medaka, and other fish, as reported in [7]. There are three kinds of IGF genes, namely, IGF1, IGF2, and IGF3, which contain highly conserved amino acid sequences. The third member of the IGF family, IGF3, was discovered around a decade ago in teleosts, and it plays an important role in regulating fish reproduction [4,6,7,8,9,10,11,12,13,14,15,16]. The production of IGFs in fish is regulated by the endocrine system, which is composed of growth hormones, somatostatin, and insulin [17,18,19,20,21,22]. Due to the scarcity of research on the roles of IGF family genes in golden pompano, more information needs to be obtained.

Somatic growth in fish and other vertebrates is primarily attained by consuming nutrients and energy readily available within their habitat’s confined range [14,23,24,25]. These nutrients are typically consumed through meals and transformed into parts of cells and tissues through cellular metabolism [25]. It is well known that nutrients and nutritional status (feeding, fasting, and refeeding) influence somatic growth in fish [24,26,27,28,29,30]. To build nutrition-related strategies to increase the yield of cultured species and evaluate the acceptability of nutrient ingredients, information on how the IGF family gene works can be utilized [7,31]. Although food and diet content significantly influence fish growth and reproduction, the growth hormone–insulin-like growth factor (GH-IGF) axis constitutes an endocrine system that regulates both somatic growth and reproduction.

The knowledge of fish reproduction is highly applicable to a fundamental understanding of fish biology [4]. The use of fish like zebrafish as a biomedical model is well considered due to their genetic similarities to humans, transparency of embryos allowing for real-time observation of development processes, rapid development, and high reproductive capacity; these characteristics enable efficient and cost-effective studies [32]. In addition, fish reproduction influences the development of aquaculture-related research, leading to increased food production worldwide [32]. Aquaculture’s global development has become more dependent on the reproduction, growth, and potential manipulation of teleost fish. Similar to somatic growth control, endogenous endocrine, neuroendocrine, and autocrine or paracrine signals work together to control fish reproduction. The role of IGF in fish reproduction is further supported by research showing that the IGF affects the production of reproductive hormones [32,33]. Studies have shown that IGFs can increase the production of testosterone, estradiol, and other hormones involved in reproduction [32,33]. IGFs can also increase the production of gonadotropin-releasing hormone (GnRH), which stimulates the reproductive hormones [11]. This is important. Still, more information is needed on the physiological functions of IGFs in golden pompano’s growth and reproductive axis.

Although IGF1, IGF2, and IGF3 have been isolated in golden pompano, and their expression patterns were examined in the regulation of different physiological processes, a comprehensive characterization of the golden pompano IGF system remains elusive. In this study, the total length of ORF cDNA was cloned to investigate the expression pattern of the IGF system in the golden pompano. We examined the mRNA level of IGF genes in several tissues; we further investigated the effects of IGF1 and IGF2 mRNA expression over fasting. Moreover, using an in vitro study, we examined the effect of E_2_ and MT on IGF family gene expression in the liver and ovary. The distinctive expression pattern of IGFs aims to provide exclusive insights into the growth, development, and reproductive processes of the golden pompano. This research seeks to contribute valuable information to enhance our comprehension of the potential markers for assessing the growth and reproduction of golden pompano, with potential applicability to other teleost fishes. In addition, the results from this study will help researchers in the field of fish physiology and endocrinology to better understand the expression of IGF family genes concerning fish growth, development, and reproduction, which will benefit the aquaculture industry.

## 2. Results

### 2.1. Molecular Cloning and Sequence Analysis

**Figure 1:**
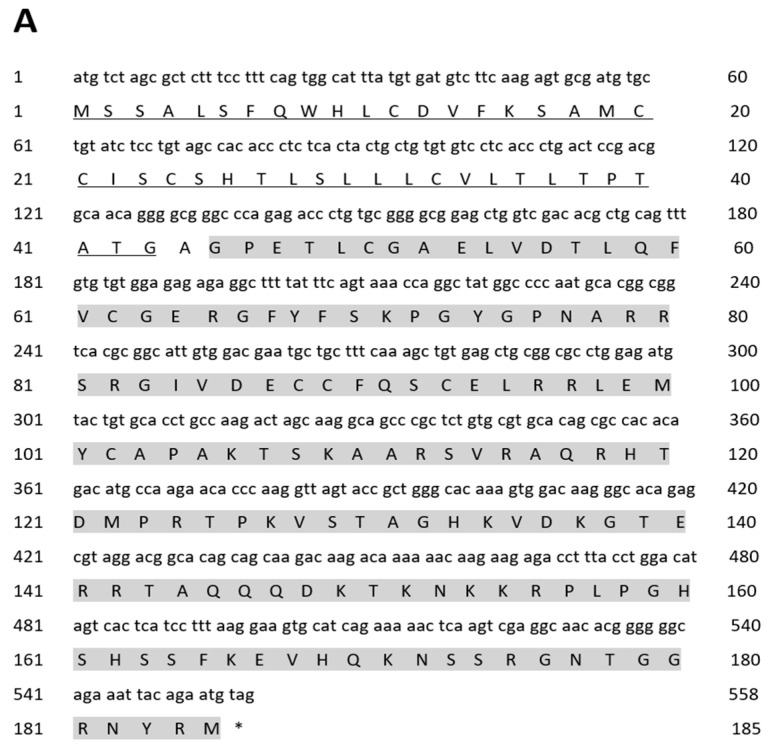

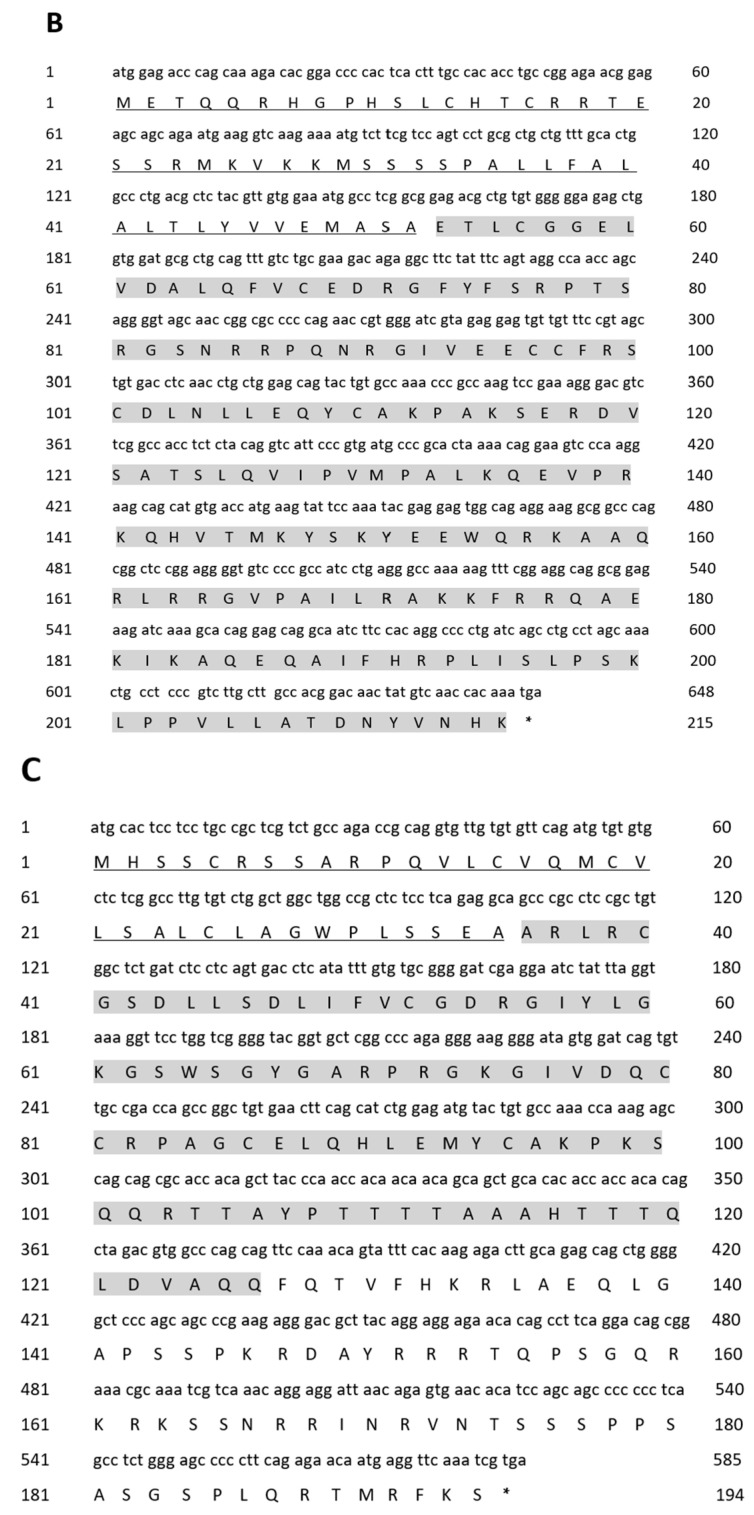
Nucleotide and deduced amino acid sequences of golden pompano IGF1 (**A**), IGF2 (**B**), and IGF3 (**C**). The signal peptide is underlined. The mature region is highlighted with grey color; the nucleotides and amino acids are numbered from **right** to **left**. The stope codon is marked by an asterisk (*).

**Figure 2:**
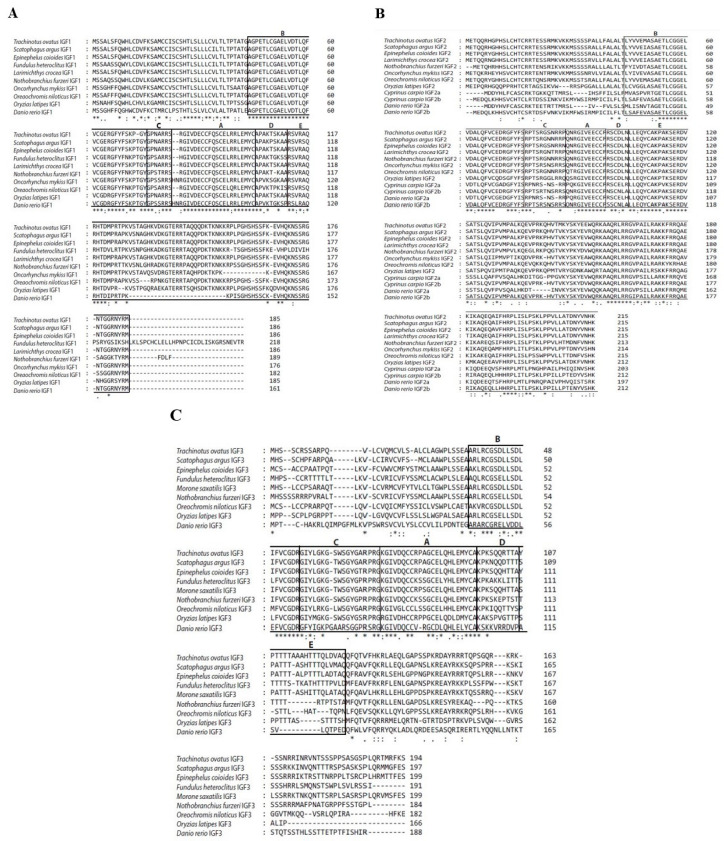
Multiple alignments of amino acid sequences of IGF1 (**A**), IGF2 (**B**), and IGF3 (**C**). Amino acid numbers are indicated on the right side of the sequences, all domains have been boxed and indicated as B, C, A, D, and E, and asterisk (*) indicates position that have fully conserved residues (identical AA residues between sequences), colon (:) indicates variation in AA at some sites (closely related AA), dot (.) indicates more than one variation in AA (distantly related AA). The DNAStar software used to construct multiple sequence alignment. See Table S1 for species name and NCBI GenBank accession numbers.

**Figure 3:**
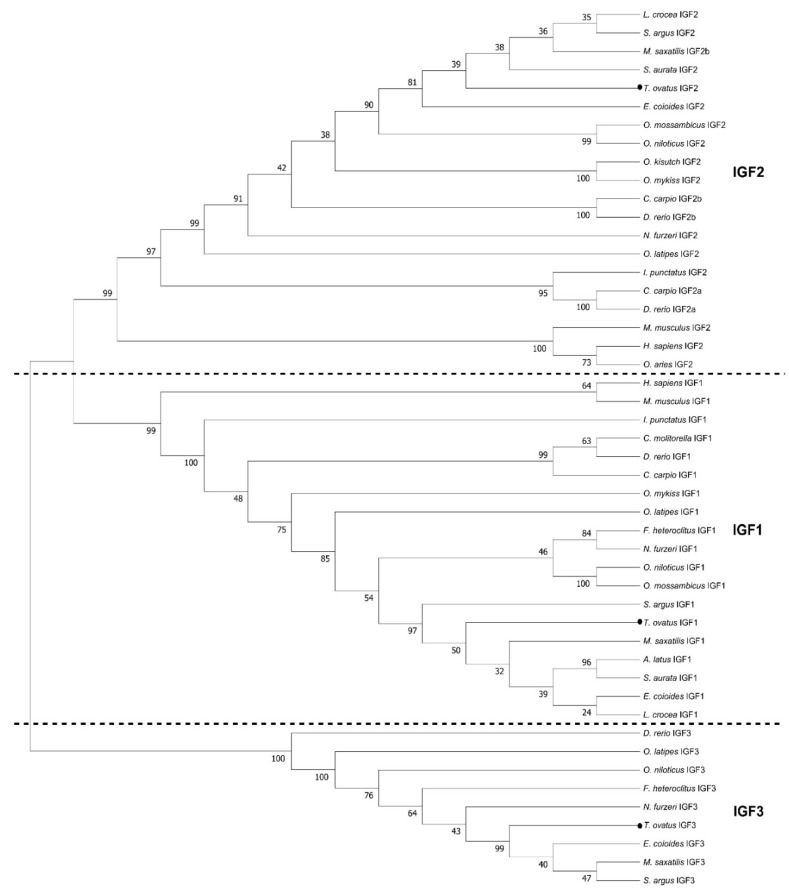
A phylogenetic tree of IGF1, IGF2, and IGF3 between golden pompano and other teleosts. The values at nodes in the system tree represent the confidence of the bootstrap test of 1000 evaluations. The evolutionary history was inferred using the neighbor-joining method. (“●”) represents *Trachinotus ovatus*, and bootstrap values (%) are indicated (1000 replicates). Amino acid sequence percentage similarity of the IGF family genes in golden pompano and other teleosts. MEGA11 was used to conduct the evolutionary analysis. See Table S1 for the species name and NCBI GenBank accession numbers.

As shown in Figure 1, the IGF family genes were cloned in the golden pompano. The IGF1 cDNA cloned from the liver mRNA contained a 558 base-pair (bp) ORF that encodes 185 amino acids (aa), which was composed of a signal peptide of 43 amino acids and a mature region of 141 amino acids (Figure 1A). The IGF2 cDNA cloned from the liver mRNA contained 648 bp ORF that encodes 215 amino acids, composed of a signa peptide of 52 amino acids and a mature region of 172 amino acids (Figure 1B). The IGF3 cDNA cloned from gonad mRNA contained a 585 bp ORF that encodes 194 amino acids, composed of a signal peptide of 35 amino acids and mature region of 91 amino acids (Figure 1C). Sequence analyses reveal that golden pompano IGF genes possessed conserved characteristics of the IGF family, like B, C, A, D, and E domains (Figure 2A–C). The IGF family gene phylogenetic tree was built using the neighbor-joining method. The analysis revealed that the *T. ovatus* IGF3 was clustered with IGF3 amino acids of selected teleosts and was independent of the IGF1 and IGF2 clades (Figure 3). The percentage similiarities of the golden pompano IGF family genes were as follows: the IGF1 had the highest similarity to *Epinephelus coioides* (98.92%) and *Larimichthys crocea* (98.92%) and the lowest similarity to *Danio rerio* (80.38%) (Table 1); IGF2 had the highest similarity to *Scatophagus argus* (96.28%) and the lowest similarity to *Cyprinus carpio* (a) (Table 2); IGF3 had the highest similarity to *Scatophagus argus* (77.60%) and the lowest similarity to *Danio rerio* (38.37%) (Table 3).

### 2.2. Tissue Distribution

**Figure 4:**
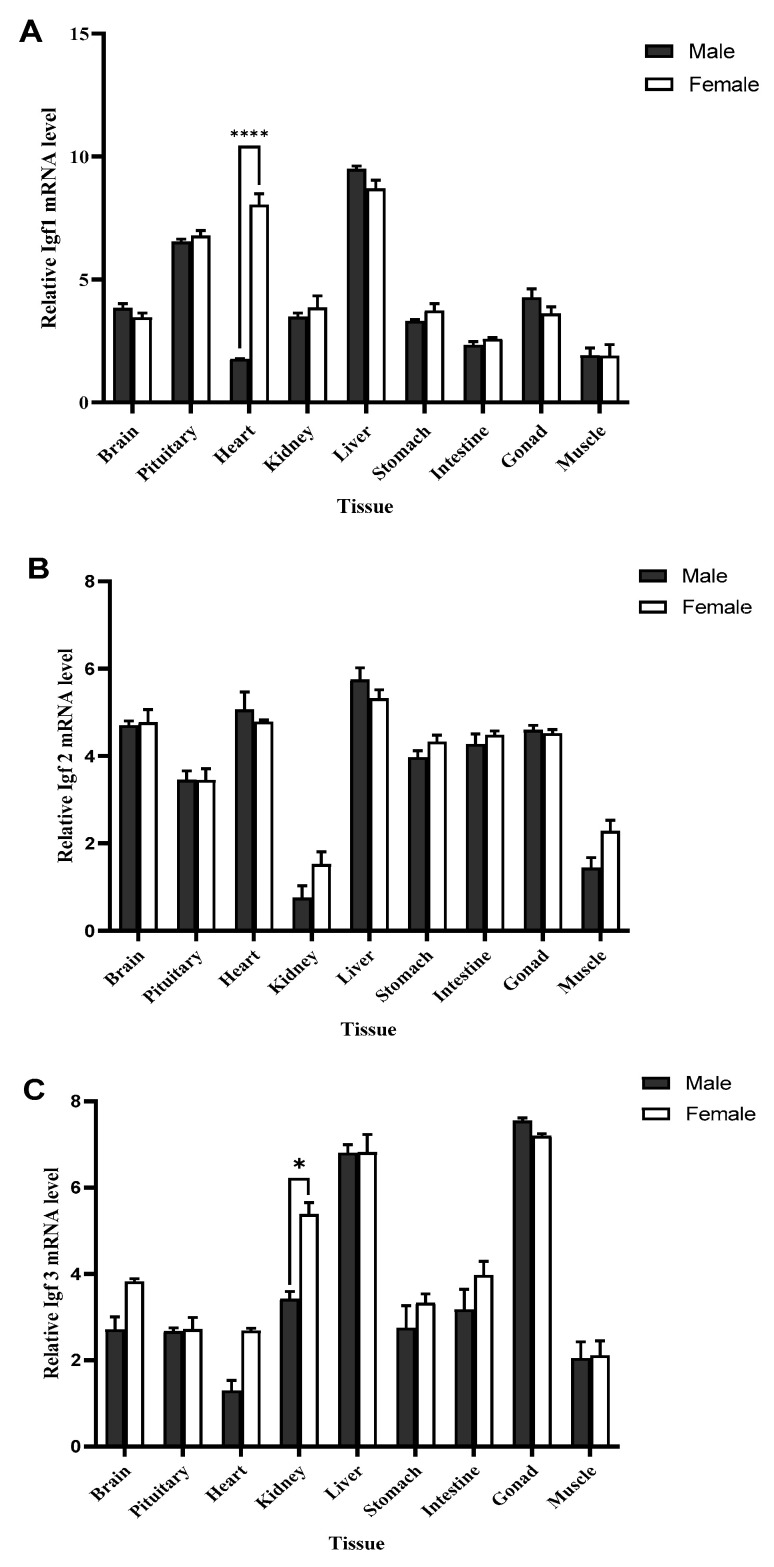
Tissue expression arrays of IGF1 (**A**), IGF2 (**B**), and IGF3 (**C**) in the golden pompano. Amplification of *β*-actin was used as the in-house regulator. The statistical significance was calculated using two-way analysis of variance (ANOVA), followed by Tukey’s test (*p* < 0.05). Data are presented as mean ± standard error (SEM). Asterisk (*) indicates a significant difference (**** *p* < 0.0001, * *p* = 0.0069).

The expression patterns of IGF1, IGF2, and IGF3 in different male and female golden pompano tissues were analyzed using quantitative real-time PCR (Figure 4). These three genes were expressed in all tissues and showed a broad tissue distribution pattern. Our analysis showed that higher levels of IGF1 mRNA in male and female golden pompano were expressed in the liver, while the lowest level was found in the muscles. The IGF1 mRNA levels were also expressed in other tissues of both male and female golden pompano, though there was a significant difference in its expression in the heart between male and female fish (Figure 4A). The IGF2 mRNA levels were highest in the liver, followed by the heart; the IGF2 mRNA level was less expressed in the kidney and muscles but evenly expressed in other tissues for both male and female golden pompano (Figure 4B). Our analysis showed that the IGF3 mRNA levels were highly expressed in gonads and followed by the liver in both male and female pompano. The IGF3 mRNA level was less expressed in the muscles and heart but evenly expressed in other tissues. Notably, a significant difference was observed in the expression level in the kidneys (Figure 4C).

### 2.3. Effects of Fasting on IGF1 and IGF2 Expression

**Figure 5:**
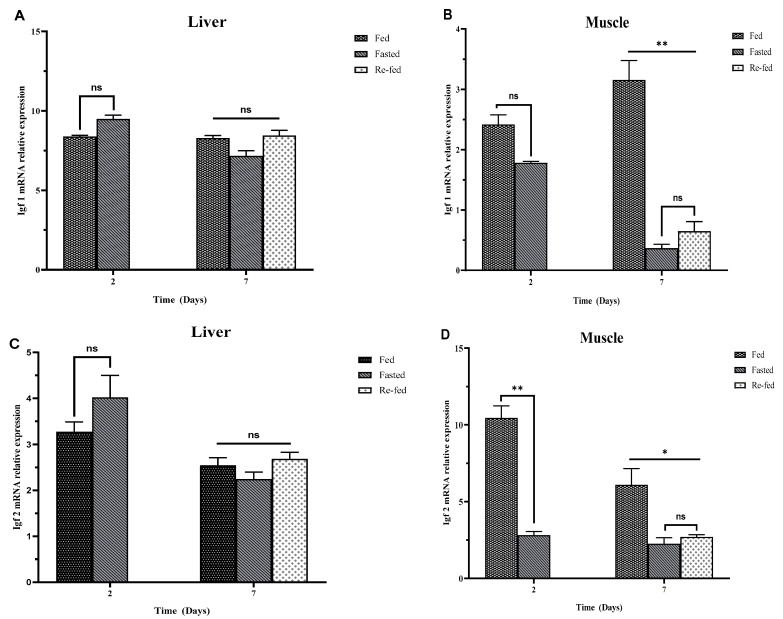
Effects of fasting on mRNA expression of IGF1 and IGF2 in the liver and muscle of golden pompano: (**A**) IGF1 in the liver, (**B**) IGF1 in muscle, (**C**) IGF2 in the liver, (**D**) IGF2 in muscle. Amplification of *β*-actin was used as the in-house regulator. Data are represented as mean ± SEM (*n* = 8). A two-way ANOVA and Turkey’s test determined significant differences (*p* < 0.05). Asterisk (*) indicates significant difference (* *p* < 0.0278, ** *p* < 0.0067). (ns) indicates nonsignificant.

The effect of fasting on the IGF gene expression in golden pompano was investigated using quantitative real-time PCR (Figure 5). In this study, the results showed no significant difference in relative IGF1 and IGF2 mRNA expression in the liver between all groups (fed, fasted, and refed) in 2 d and 7 d, as shown (*p* > 0.05; Figure 5A,C). In addition, the results indicated that there was no significant difference in relative muscle IGF1 mRNA levels between the fed and fasted group in 2 d (*p* > 0.05; Figure 5B), but there was a significant difference between fed, fasted, and refed groups in 7 d (*p* < 0.05, Figure 5B). However, there was no significant difference between fasted and refed groups in 7 d. There was a significant difference in relative muscle IGF2 mRNA expression in the fed, fasted, and refed groups in 2 d and 7 d. No significant difference between fasted and refed groups in 7 d is shown in Figure 5D.

### 2.4. In Vitro Effects of E_2_ on IGFs mRNA Expression in the Liver and Gonads of Golden Pompano

**Figure 6:**
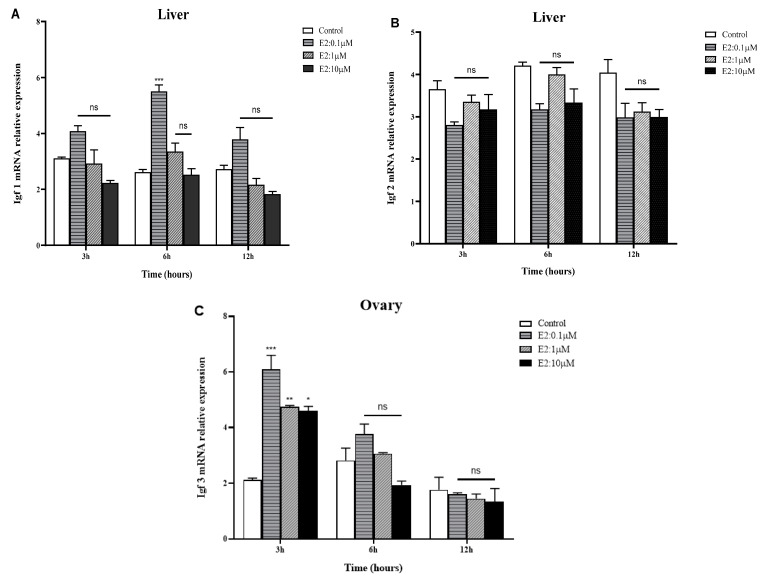
Time and dose-dependent in vitro effects of E_2_ on mRNA expression of IGF genes in the liver and ovary of golden pompano: (**A**) IGF1, (**B**) IGF2, and (**C**) IGF3. Amplification of *β*-actin was used as the in-house regulator. Data are presented as mean ± standard error (SEM) (*n* = 3), and the statistical significance (compared to the control group) was determined using two-way ANOVA, followed by Tukey’s test (*p* < 0.05). (*) indicates significant differences (* *p* < 0.01, ** *p* < 0.0025, *** *p* < 0.0005). (ns) indicates nonsignificant.

Figure 6 summarizes the effects of E_2_ on the mRNA expression levels of three IGF genes in golden pompano (male for IGF1 and IGF2, female for IGF3). In the liver, incubation of E_2_ was observed to decrease the steady-state levels of IGF1 mRNAs in a dose-dependent manner. Significantly, the minimum IGF1 mRNA expression level observed at 12 h was 10 μM, with a maximum expression level at 6 h (0.1 μM). There was no significant difference in IGF1 mRNA expression at 3 h and 12 h in a dose-dependent manner (Figure 6A). On the other hand, there were no significant differences in the expression levels of liver IGF2 mRNA after E_2_ in vitro incubation at 3, 6, and 12 h in a dose-dependent manner (Figure 6B). In the ovary, the IGF3 mRNA expression level was higher at 3 h (at 0.1 μM), but the expression level was significantly decreased at 3 h (at 1 and 10 μM); similar to IGF1, the general observation shows that the IGF3 mRNA expression level was significantly decreased in a dose- and time-dependent manner (Figure 6C). Additionally, the maximum expression of IGF3 mRNA was observed at 3 h (0.1 μM) and significantly declined at 6 h, reaching the minimum level at 12 h in a dose-dependent manner.

### 2.5. In Vitro Effects of MT on IGF mRNA Expression in the Liver and Gonads of Golden Pompano

**Figure 7:**
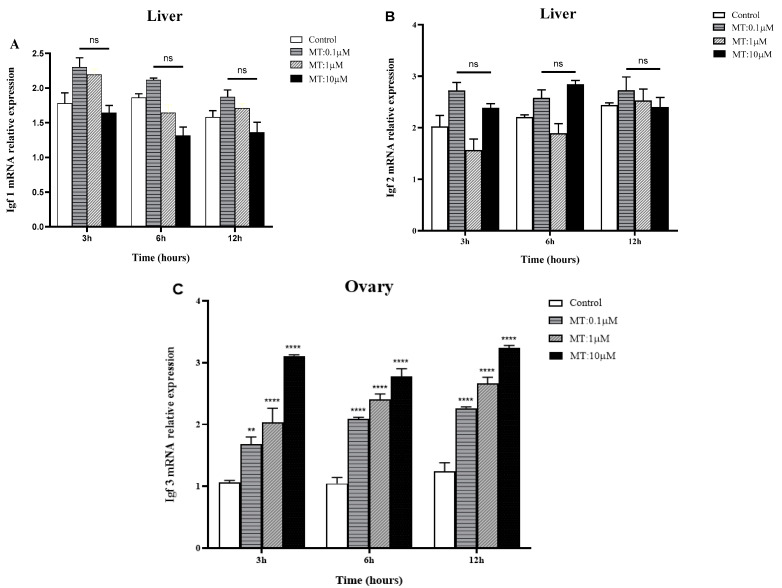
Time and dose-dependent in vitro effects of MT on mRNA expression of IGF genes in the liver and ovary of golden pompano: (**A**) IGF1, (**B**) IGF2, and (**C**) IGF3. Amplification of *β*-actin was used as the in-house regulator. Data are presented as mean ± standard error (SEM) (*n* = 3), and the statistical significance (compared to the control group) was determined using two-way ANOVA, followed by Tukey’s test (*p* < 0.05). (*) indicates significant differences (** *p* < 0.0021, **** *p* < 0.0001). (ns) indicates nonsignificant.

The effect of MT on mRNA expression in the liver and ovary of three IGF genes is summarized in Figure 7. The results show that there was no significant difference in the IGF1 mRNA expression level in a time- and dose-dependent manner at 3 h, 6 h, and 12 h at different concentrations (0.1, 1, and 10 μM) (see Figure 7A). The maximum IGF1 mRNA level was observed at 3 h (0.1 µM), with a minimum expression level at 6 h (10 µM). The IGF2 mRNA expression levels were observed to be similar to IGF1 mRNA expression, with slight differences. The maximum expression level was at 6 h (0.1 µM), and the minimum expression level was observed at 3 h (1 µM). At 3 h and 6 h, the IGF2 mRNA expression level trend was similar and highly expressed at 0.1 µM and 10 µM while it declined at 1 µM (see Figure 7B). On the other hand, the IGF3 mRNA level was significantly increased from lower to higher doses (0.1, 1, and 10 μM) of MT in a time-dependent manner (Figure 7C).

## 3. Discussion

In the present study, the ORF cDNA sequence of the IGF family genes was cloned from the golden pompano. The golden pompano deduced amino acids of IGF1 showed high sequence identity with other species, *E. coioides* and *L. crocea,* while the golden pompano IGF2 showed a high sequence identity with *S. argus*. The pompano IGF3 also showed a high amino acid sequence identity with *S. argus*. The structure of the golden pompano and other teleost IGFs is highly conserved through evolution [12,34], because fish are the largest and most diverse group of vertebrates and exhibit the highest levels of evolutionary constraints on different IGF sequences, indicating the importance of these peptides on growth, development, and reproduction [3,4,31,35,36]. In addition, the highly conserved sequences of all known IGF orthologues imply that the relationship between each protein’s structure and function is also conserved in vertebrates.

The tissue distribution experiment showed that the IGF mRNA was highly expressed in the liver, the primary source of endocrine IGF1 and IGF2 in golden pompano and other vertebrates [7,37,38]. Apart from being highly expressed in the liver, both IGF1 and IGF2 expression has been observed in various tissues in several fish species, including brains, pituitary, hypothalamus, gills, heart, intestines, stomach, spleens, and gonads, with the lowest expression in muscles and the kidney. Unlike mammals, where IGF2 is found in significant concentrations in many fetal tissues but rapidly declines during early postnatal development, the IGF2 level is highly expressed in teleosts after birth [15,39,40,41,42,43,44]. Our results showed that IGF3 is highly expressed in the gonads and liver, while there was a lower expression in the heart and muscle as well as in other tissues for both male and female golden pompano. The predominant expression of IGF3 in gonads and the liver suggests its significant role in fish reproduction, as previously reported in [4,45,46,47,48]. IGFs are likely expressed in a range of tissues in golden pompano, suggesting that they may have other functions in addition to promoting growth, development, and reproduction, as initially theorized [10,11,12,23].

The effects of fasting on IGF expression in fish have been studied in different contexts. In recent years, the effects of fasting on insulin-like growth factor expression in fish have become a topic of interest for researchers. Studying the effect of short-term and long-term fasting is essential for understanding the regulation of the metabolism and somatic growth in fish. In the present study, the IGF1 and IGF2 mRNA expression levels in golden pompano’s liver had no significant difference after short-term (2 days) and long-term (7 days) fasting. The IGF1 mRNA expression in the muscles reduced after long-term fasting, but it increased after refeeding. Previous reports have indicated that hepatic IGF1 mRNA expression is decreased after fasting and recovered by refeeding in Coho salmon [49], hybrid striped bass [50], tilapia [51], European sea bass [52], Yellowtail [25], and channel catfish [53,54]. The IGF2 mRNA expression in muscles decreased after short-term and long-term fasting, suggesting that muscles are susceptible even to short-term fasting compared to the liver. The IGF1 and IGF2 mRNA levels did not approach the original levels after refeeding on the seventh day, especially in the muscles, indicating that a more extended period is required to recover fully. In sea bass, the IGF1 and IGF2 mRNA levels reveal different gene regulations and distinct roles in its growth [50]. Research indicates that prolonged food restriction lowers the hepatic IGF1 mRNA levels in several fish species [39,55]. In Nile tilapia, the IGF1 and IGF2 mRNA levels were lower in starved fish than in fed fish [51]. Additionally, several studies have shown that the availability of nutrients and a wide range of environmental conditions influence the rate of muscle growth in fish [3,17,24].

In addition, a decrease in IGF expression due to the lack of food availability decreased insulin and glucagon levels [3,44,56,57,58,59]. Studies on zebrafish, rainbow trout, and juvenile tilapia showed that the effects of fasting on IGF1 and IGF2 mRNA expression caused a decrease in IGF expression in the liver, kidney, and muscle tissues [9,10,30,52,53,60,61]. The decrease in IGF expression was most pronounced in the liver, muscles, and kidney tissues, suggesting these tissues are more sensitive to the effects of fasting. In addition, the effects of fasting on IGF expression in fish have been studied in relation to environmental stressors [24]. For example, in Atlantic salmon, fasting caused a decrease in IGF expression in the liver, kidney, and muscle tissues [58,62]. The decrease in IGF expression may have implications for the health and well-being of fish, since these genes tend to regulate metabolism, growth, development, and reproduction.

The results from this study show that both E_2_ and MT significantly affect the expression of IGF genes in golden pompano in both a dose- and time-dependent manner, similar to other studies; both E_2_ and MT had a regulatory effect on IGF1 and IGF2 mRNA expression [63]. E_2_ has been shown to reduce the expression of IGF3 in rainbow trout [64]. MT has been shown to regulate the expression of IGFs in *S. aurata* [65,66]. In addition, other research indicates that E_2_ and MT tend to regulate gene expression in teleosts [67]. The effect of different concentrations of E_2_ and MT on IGF1 and IGF2 were measured in both zebrafish and juvenile tilapia. The study revealed the stimulatory effect of E_2_ on IGFs, while MT exhibited an inhibitory effect [18,47]. In common carp [66], the results showed that E_2_ and MT significantly affected IGF2 expression, with E_2_ increasing IGF2 mRNA levels and MT decreasing IGF2 mRNA levels [66,68]. The study concluded that E_2_ and MT had opposite effects on IGF2 mRNA expression in the carp and that E_2_ had a more pronounced effect than MT [66,68]. Another study on the Coho salmon showed that both E_2_ and MT had a positive effect on IGF2 mRNA expression [69]. The results of these studies suggest that E_2_ and MT have different effects on IGF mRNA expression in fish.

In addition, there are several observations that suggest the ability of E_2_ to regulate peripheral components of the GH-IGF system [65,66]. One such observation is that E_2_ has been shown to reduce the steady-state levels of mRNAs that encode GHR in various tissues [66]. In contrast, the effects of E_2_ and MT on IGFs, particularly IGF3 in fish, are complex and largely unexplored. To the best of our understanding, this study represents the first examination of the impact of sex steroids on IGF3 expression, suggesting that IGF3 may indeed be regulated by sex steroids. Given the limited literature on the effects of E_2_ and MT sex steroids on IGF gene expression, this study contributes to our understanding of their potential on the IGF family genes expression in golden pompano, particularly concerning fish reproduction. Treatment with E_2_ and MT may either stimulate or inhibit IGF mRNA expression. However, our results do not conclusively confirm the inhibitory mechanism of sex steroids on IGF mRNA expression. Further research is needed to explore the gender-specific differences of E_2_, MT, and IGF mRNA expression to enhance our understanding of these relationships in fish.

## 4. Materials and Methods

### 4.1. Experimental Fish Collection

In this study, the experimental fish collection was carefully organized to facilitate various experiments, including those focused on tissue distribution, the effects of fasting and refeeding, and the effects of E_2_ and MT in vitro incubation on IGF family gene expression. In all experiments, a total of 64 fish were utilized, with consideration given to gender balance and experimental requirements. Fish were purchased from the Zhanjiang, Guangdong, China, aquatic product market and then transported to the experiment room at Guangdong Ocean University. A total of 40 fish for the effect of the fasting and refeeding experiment were cultured at Guangdong Havwii Fry Cultivation Base (Zhanjiang, Guangdong, China). Fish were carefully selected based on their weight, ensuring consistency and reliability across experiments, as explained in their respective sections (Section 4.4, Section 4.5 and Section 4.6). Before sampling, all fish were anesthetized in a solution with 50 mg/L of tricaine methane sulfonate (MS-222, Sigma, Saint Louis, MO, USA). In preparation for ribonucleic acid (RNA) extraction, tissues were snap-frozen in liquid nitrogen and kept at −80 °C.

The Animal Research and Ethics Committees of the Fisheries College of Guangdong Ocean University, Zhanjiang, Guangdong, China, approved all experiments in this study (Ethical batch number: GDOU-IACUC-2021-A1229).

### 4.2. RNA Extraction and cDNA Synthesis

RNA was extracted from brain, pituitary, heart, kidney, liver, stomach, intestine, gonads, and muscle using the Trizol reagent kit (Takara, Dalian, China). The integrity of RNA was determined on a 1% agarose electrophoresis gel with ethidium bromide staining. RNA’s purity and yield were measured using a NanoDrop 2000C spectrophotometer (ThermoScientific, Waltham, MA, USA). Total RNA was reverse transcribed with super script protocol. The first strand of cDNA was synthesized using the Prime ScriptTM RT Reagent Kit with gDNA Eraser (RR047A; Takara Bio, Dalian, China), a mixture of 20 µL, made up of 15 µL (1 µg RNA + RNase free water), 4 µL of all-in-one supermix for qPCR, and 1 µL of gDNA remover was added and incubated at 50 °C for 15 min and 85 °C for 5 min. The final concentration was diluted three times to give a total volume of 80 µL cDNA for use as templates in all PCR reactions.

### 4.3. Molecular Cloning and Sequence Analysis

The cDNA sequence was obtained from the transcriptome data of pompano (NCBI Sequence Read Archive (SRA) database: IGF1 (MH300904.1 and KT727922.1), IGF2 (MH300905.1 and KT727923.1), and IGF3 (MH300906.1). Subsequently, we designed primers to the pompano IGF1, IGF2, and IGF3 gene (Table 4) using Primer Premier 5.0 software, and the IGF cDNA was cloned from the cDNA, which was reverse-transcribed from the total RNA of liver and gonads. PCR Thermal Cycler (BIO-RAD-C1000, Hercules, CA, USA) was used to amplify genes. The PCR products were verified by 1.5% agarose gel electrophoresis. The target fragments were extracted and cloned into *pEASY*-T3 vectors (TransGen Biotech, Beijing, China). Positive clones were sequenced by Sango Biotech (Shanghai, China).

The ORF of pompano IGF1, IGF2, and IGF3 were predicted, and the ORF amino acid (aa) sequence was translated using DNAstar software 17. SignalP (https://services.healthtech.dtu.dk/services/SignalP-6.0/, accessed on 13 September 2023), was used to identify a putative signal peptide. NeuroPred (http://stagbeetle.animal.uiuc.edu/cgi-bin/neuropred.py, accessed on 14 September 2023), was used to predict the cleavage sites of the peptide precursors. Functional sites of proteins were predicted using Softberry (http://www.softberry.com/, accessed on 14 September 2023). The cloned IGF1, IGF2, and IGF3 sequences were submitted to the NCBI Sequence Read Archive (SRA) database: OR038159, OR038160, and OR038161, respectively. Multiple alignments of the Nicol1 amino acid sequences were then performed using DNAstar software. Finally, a phylogenetic tree was constructed using the neighbor-joining method in MEGA 11.0 software.

### 4.4. Tissue Distribution of IGF Family Genes

To determine IGF family gene tissue distribution, the total RNA was extracted from 100 mg of different tissues from 12 golden pompano (6 males and 6 females) (Table S2), including the brain, pituitary, heart, kidney, liver, stomach, intestine, gonads, and muscles, by following the same procedures mentioned in Section 4.2, above. Tissue distribution was analyzed by reverse-transcription quantitative real-time PCR (RT-qPCR). RT-qPCR was performed using a CFX96 Real-Time PCR Detection System (Bio-Rad, Indianapolis, IN, USA). *β*-actin was used as an internal control. We designed a pair of IGF1, IGF2, IGF3, and *β*-actin primers for tissue distribution based on cloned sequence to evaluate the expression of genes in selected tissues (see Table 1). The amplification regime consisted of 35 cycles of 30 s at 94 °C, 60 °C for 30 s, and 72 °C for 30 s, followed by further amplification at 72 °C for 10 min. PCR products were separated using 1.5% agarose gel.

### 4.5. Effects of Fasting on IGF1 and IGF2 Expression in the Liver and Muscles

The fish fasting and refeeding experiment was conducted in September and October to investigate the expression of IGF1 and IGF2 in response to varying nutritional status. A total of 40 golden pompano (20 males and 20 females) with 100–120 g body weight were randomly divided into five groups (*n* = 8) and placed into five 1 m^3^ (~3.18 m diameter and 1 m height) circular seawater tanks with a constant water supply, at a temperature of 29–31 °C, with constant supply of oxygen using aerators; during this experiment, fish were maintained under ambient photoperiod with 12 h of daylight and 12 h of darkness on flow-through filtered seawater. Water quality parameters, including dissolved oxygen, pH, temperature, and ammonia, were measured using an HQ30d (HACH30d, Loveland, CO, USA). This experiment was designed based on previous report [29]. Commercial meals from (YueQun Ocean Biological Research Development Company, Jieyang, China) at a ration of ~2% body weight (BW)/meal were used to feed fish daily at 9:00 a.m. After two weeks of acclimatization, two groups of fish were fed daily for 2 or 7 days (d), while the other three groups remained starved. Fish from the fed and fasted groups were sampled after 2 days. At the end of the 7-day starvation, one of the fasting groups was refed to satiety. Then, the fed, fasted, and refed groups were sampled at 3 h after the scheduled feeding time. The liver and muscles of each fish were removed and stored in the sample stabilizer (Acrexen), then stored overnight at 4 °C to allow sufficient penetration of the sample to stabilize into the sample tissues before transferring the sample to −20 °C. Subsequently, total RNA extraction was performed.

### 4.6. In Vitro Incubation of Golden Pompano with E_2_ and MT on IGF Gene Expression

To study the effects of E_2_ and MT on IGF gene expression, absolute ethanol was used to dissolve E_2_ and MT steroids. A total of 12 fish (6 males and 6 females), averagely weighing 450 g as reported by [1], were anesthesized with MS-222; then, their liver and gonads were carefully dissected, washed three times, and placed in a petri dish containing an M199 and L15 medium, respectively. The tissue fragments were transferred onto 24-well culture plates for pre-incubation. After 2 h of pre-incubation at 25–28 °C with 5% CO_2_, the medium was removed and replaced with a fresh medium containing 0.1, 1, and 10 μM of E_2_/MT. The experimental and control groups were treated at the same time. After 3, 6, and 12 h of incubation, the liver and gonad fragments were collected with extracted RNA. Quantitative real-time PCR was used to measure their relative expression with specific primers, as decribed in Section 4.7.

### 4.7. Quantitative Real-Time PCR (RT-qPCR) Analysis

We determined the mRNA level of IGFs in selected golden pompano tissues, the effects of long-term fasting and refeeding on the expression of the IGF genes, and the in vitro incubation of golden pompano liver and gonads with E_2_ and MT using quantitative real-time PCR (RT-qPCR). The primers were designed using Primer Premier 5.0 software based on ORF of the cloned full-length cDNA sequence of IGF family genes (see Table 1). RT-qPCR was performed in triplicate for each sample on a CFX96 Real-Time PCR Detection System (Bio-Rad, Indianapolis, IN, USA) in 20 µL reactions containing the following components: 2 µL cDNA (template), 10 µL SYBR Premix EXTaq (TaKaRa, Shiga, Japan), 0.8 µL of each primer (forward and reverse), and 6.4 µL RNase-free water. The PCR parameters were 40 cycles at 95 °C for 5 s, 60 °C for 30 s, and a dissociation curve analysis of 5 s per step from 65 to 95 °C. All samples for relative mRNA level analysis were run once in a single assay. Relative expression was calculated by the *R* = 2^−∆∆*Ct*^ method, and the data were presented as mean ± standard error (mean ± SEM).

### 4.8. Statistical Analysis

All statistical analyses were performed using Statistical Package for the Social Sciences (SPSS) 25.0 (SPSS, Chicago, IL, USA). Significant differences in the data among groups were determined at some point by the two-way analysis of variance (ANOVA), followed by Tukey’s multiple comparison tests. A likelihood level below 0.05 (*p* < 0.05) was used to specify significance.

## 5. Conclusions

Three IGF family genes were identified in the golden pompano; their structures are highly conserved compared to other teleosts. The tissue distribution patterns suggest that these genes could be involved in various physiological processes, including somatic growth, metabolism, and reproduction. The effect of short-term and long-term fasting suggests that IGF1 and IGF2 support muscle and liver compensatory development through refeeding. The effects of sex steroids (E_2_ and MT) have been shown to regulate the expression of IGFs in golden pompano. Further experiments on the effects of fasting under environmental stressors and the effect of sex steroids on IGF expression for all sexes need to be explored, and this pattern must be confirmed in other teleosts. Furthermore, the findings of this study will assist researchers in the realms of fish physiology and endocrinology in gaining a more comprehensive understanding of the expression of IGF family genes in relation to fish growth, development, and reproduction. Such insights are expected to have positive implications for the aquaculture industry.

## Figures and Tables

**Table 1:** Percentage identity matrix of amino acids for the selected teleost IGF1.

	1	2	3	4	5	6	7	8	9	10
1: *Trachinotus ovatus*	100									
2: *Scatophagus argus*	96.76	100								
3: *Epinephelus coioides*	98.92	97.85	100							
4: *Fundulus heteroclitus*	80.87	80.98	80.98	100						
5: *Larimichthys crocea*	98.92	97.85	100	80.98	100					
6: *Nothobranchius furzeri*	88.59	87.57	88.65	82.89	88.65	100				
7: *Oncorhynchus mykiss*	89.02	87.36	89.08	73.99	89.08	82.66	100			
8: *Oreaochromis niloticus*	88.95	89.56	90.11	79.44	90.11	87.29	85.38	100		
9: *Oryzias latipes*	86.89	84.78	86.96	76.50	86.96	84.25	80.81	82.22	100	
10: *Danio rerio*	80.38	77.99	79.87	68.79	79.87	77.22	86.67	78.48	78.34	100

**Table 2:** Percentage identity matrix of amino acid for the selected teleost IGF2.

	1	2	3	4	5	6	7	8	9	10	11	12
1: *Trachinotus ovatus*	100											
2: *Scatophagus argus*	96.28	100										
3: *Epinephelus coioides*	94.88	95.81	100									
4: *Larimichthys crocea*	95.35	97.21	95.35	100								
5: *Nothobranchius furzeri*	84.04	85.45	84.98	84.51	100							
6: *Oncorhynchus mykiss*	84.11	84.58	83.64	85.05	78.30	100						
7: *Oreaochromis niloticus*	89.77	90.70	89.30	90.23	82.16	80.84	100					
8: *Oryzias latipes*	75	74.53	75	74.06	68.10	68.72	69.81	100				
9: *Cyprinus carpio (a)*	52.71	53.20	53.69	53.69	52.22	54.68	53.69	46	100			
10: *Cyprinus carpio (b)*	77.36	78.77	78.77	79.72	75.47	75.94	74.06	63.16	54.68	100		
11: *Danio rerio (a)*	55.84	56.35	56.85	57.36	55.84	58.88	56.35	47.42	83.25	55.84	100	
12: *Danio rerio (b)*	77.36	78.77	78.77	79.72	75.47	76.89	74.06	63.16	54.19	95.28	55.33	100

**Table 3:** Percentage identity matrix of amino acid for the selected teleost IGF3.

	1	2	3	4	5	6	7	8	9
1: *Trachinotus ovatus*	100								
2: *Scatophagus argus*	77.60	100							
3: *Epinephelus coioides*	72.02	74.62	100						
4: *Fundulus heteroclitus*	59.78	62.77	59.47	100					
5: *Morone saxatilis*	77.20	80.20	73.87	64.21	100				
6: *Nothobranchius furzeri*	64.77	67.78	63.74	69.78	66.48	100			
7: *Oreaochromis niloticus*	59.09	60.00	59.89	54.24	61.54	57.31	100		
8: *Oryzias latipes*	56.88	52.96	51.52	51.22	52.73	53.75	47.83	100	
9: *Danio rerio*	38.37	38.07	39.89	36.52	37.64	37.64	33.73	38.61	100

**Table 4:** Primers used in this study.

Primer Name	Sequence (5′-3′)	Application	Reference
IGF1 F	GACCCGTGGGGATGTCTAGC	Cloning of ORF	Designed by author
IGF1 R	ACAGAATGTAGGGAAGGAGC	Cloning of ORF	Designed by author
IGF2 F	GACTACTGCCATCTGACATG	Cloning of ORF	Designed by author
IGF2 R	GCACAGACAAGAGTTTGGAG	Cloning of ORF	Designed by author
IGF3 F	GATGCACTCCTCCTGCCGCTC	Cloning of ORF	Designed by author
IGF3 R	CGTGACCTCTGACCTCATGGCC	Cloning of ORF	Designed by author
IGF1 F	TACTGCTGTGTGTCCTCAC	qPCR	Designed by author
IGF1 R	CGCCTGGAGATGTACTGTGCA	qPCR	Designed by author
IGF2 F	TCGGCGGAGACGCTGTGTG	qPCR	Designed by author
IGF2 R	TTCCCGTGATGCCCGCACTA	qPCR	Designed by author
IGF3 F	GGGGATCGAGGAATCTATT	qPCR	Designed by author
IGF3 R	AGTTCCAAACAGTATTTCACAA	qPCR	Designed by author
*β*-actin F	GAGAGGTTCCGTTGCCCAGAG	qPCR	[2]
*β*-actin R	CAGACAGCACAGTGTTGGCGT	qPCR	[2]

## Data Availability

Data are contained within the article.

## Supplementary Materials

The following supporting information can be downloaded at: https://www.mdpi.com/article/10.3390/ijms25052499/s1.
