# Supplementary material for: Identification of Insulin-like Growth Factor (IGF) Family Genes in the Golden Pompano, Trachinotus ovatus: Molecular Cloning, Characterization and Gene Expression
Source: Int J Mol Sci. 2024 Feb 21;25(5):2499. doi: 10.3390/ijms25052499 (PMC10931442; doi:10.3390/ijms25052499)
Supplement: Supplementary file 1 [file ijms-25-02499-s001.zip › ijms-2807866-supplementary.pdf]

Supplementary materials

Table S1. List of species and their NCBI IDs used in this study

SN	Gene	Specie name	NCBI_Accession ID
1	IGF1	*Acanthopagrus latus*	AAT35826.1
2	IGF1	*Cyprinus carpio*	AAP78926.1
3	IGF1	*Cirrhinus molitorella*	AAY21902.1
4	IGF1	*Epinephelus coioides*	AMR58932.1
5	IGF1	*Fundulus heteroclitus*	JAQ23103.1
6	IGF1	*Homo sapiens*	AAA52789.1
7	IGF1	*Ictalurus punctatus*	AAZ28918.1
8	IGF1	*Larimichthys crocea*	TMS03093.1
9	IGF1	*Mus musculus*	AAH12409.1
10	IGF1	*Morone saxatilis*	XP_035535299.1
11	IGF1	*Nothobranchius furzeri*	AGY80124.1
12	IGF1	*Oncorhynchus kisutch*	XP_020361825.1
13	IGF1	*Oncorhynchus mykiss*	NP_001118168.1
14	IGF1	*Oreochromis niloticus*	ABY88872.1
15	IGF1	*Oreochromis mossambicus*	AAC17494.1
16	IGF1	*Oryzias latipes*	ATY35168.1
17	IGF1	*Sparus aurata*	AAY46225.1
18	IGF1	*Scatophagus argus*	QBG05668.1
19	IGF1	*Trachinotus ovatus*	QDD55744.1
20	IGF1	*Danio rerio*	AAI14263.1
21	IGF2	*Epinephelus coioides*	AMR58927.1
22	IGF2	*Homo sapiens*	AAB34155.1
23	IGF2	*Ictalurus punctatus*	ADO29240.1
24	IGF2	*Larimichthys crocea*	XP_019124122.1
25	IGF2	*Mus musculus*	AAH58615.1
26	IGF2	*Nothobranchius furzeri*	KAF7224778.1
27	IGF2	*Ovis aries*	AAB60626.1
28	IGF2	*Oncorhynchus kisutch*	XP_020311888.1
29	IGF2	*Oncorhynchus mykiss*	NP_001118169.1
30	IGF2	*Oreochromis mossambicus*	AAC17496.1
31	IGF2	*Oreochromis niloticus*	ABY88873.1
32	IGF2	*Oryzias latipes*	XP_023811944.1
33	IGF2	*Sparus aurata*	XP_030281828.1
34	IGF2	*Scatophagus argus*	QBM11764.1
35	IGF2	*Trachinotus ovatus*	QDD55745.1
36	IGF2a	*Cyprinus carpio*	ADQ48003.1
37	IGF2a	*Danio rerio*	NP_571508.1
38	IGF2b	*Cyprinus carpio*	XP_018967677.2
39	IGF2	*Morone saxatilis*	XP_035520505.1
40	IGF2	*Danio rerio*	NP_001001815.1
41	IGF3	*Epinephelus coioides*	AML84199.1
42	IGF3	*Fundulus heteroclitus*	XP_035985858.1

43	IGF3	*Morone saxatilis*	XP_035513051.1
44	IGF3	*Nothobranchius furzeri*	SBP52008.1
45	IGF3	*Oncorhynchus mykiss*	XP_021469905.2
46	IGF3	*Oreochromis niloticus*	NP_001266565.1
47	IGF3	*Oryzias latipes*	QGX02415.1
48	IGF3	*Scatophagus argus*	UKD40691.1
49	IGF3	*Trachinotus ovatus*	QDD55746.1
50	IGF3	*Danio rerio*	NP_001108522.1

Table S2: Number of fish obtained for various experiments

Experiment Name	Male fish used	Female fish used	Weight (g)	Total fish
**Tissue distribution**	6	6	500-1000	12
**Feeding, fasting, and refeeding**	20	20	100-120	40
***In vitro* incubation of E2 and MT**	6	6	450	12
**Total**				64

Table S3. P- value for statistical tests comparing different groups for all graphs.

Table S3: Presents P values for Panel A-Tissue distribution

Tukey's multiple comparisons test	Mean Diff.	95.00% CI of diff.	Significant?	Summary	Adjusted P Value
Brain:Male vs. Brain:Female	0.374	-1.36 to 2.11	No	ns	>0.9999
Pituitary:Male vs. Pituitary:Female	-0.245	-1.98 to 1.49	No	ns	>0.9999
Heart:Male vs. Heart:Female	-6.27	-8.01 to -4.53	Yes	****	<0.0001
Kidney:Male vs. Kidney:Female	-0.371	-1.57 to 0.826	No	ns	0.9953
Liver:Male vs. Liver:Female	0.792	-0.946 to 2.53	No	ns	0.8892
Stomach:Male vs. Stomach:Female	-0.427	-2.17 to 1.31	No	ns	0.9996
Intestine:Male vs. Intestine:Female	-0.247	-1.99 to 1.49	No	ns	>0.9999
Gonad:Male vs. Gonad:Female	2.33	0.593 to 4.07	Yes	**	0.0039
Muscle:Male vs. Muscle:Female	-1.32	-3.06 to 0.417	No	ns	0.256

Table S3: Presents P values for Panel B-Tissue distribution

Tukey's multiple comparisons test	Mean Diff.	95.00% CI of diff.	Significant?	Summary	Adjusted P Value
Brain:Male vs. Brain:Female	-0.0716	-1.10 to 0.952	No	ns	>0.9999
Pituitary:Male vs. Pituitary:Female	0.00169	-1.02 to 1.03	No	ns	>0.9999
Heart:Male vs. Heart:Female	0.278	-0.746 to 1.30	No	ns	0.9989
Kidney:Male vs. Kidney:Female	-0.768	-1.79 to 0.256	No	ns	0.2714
Liver:Male vs. Liver:Female	0.434	-0.590 to 1.46	No	ns	0.9319
Stomach:Male vs. Stomach:Female	-0.356	-1.38 to 0.668	No	ns	0.9861
Intestine:Male vs. Intestine:Female	-0.207	-1.23 to 0.817	No	ns	>0.9999

Gonad:Male vs. Gonad:Female	0.083	-0.941 to 1.11	No	ns	>0.9999
Muscle:Male vs. Muscle:Female	-0.842	-1.87 to 0.182	No	ns	0.173

Table S3: Presents P values for Panel C-Tissue distribution

Tukey's multiple comparisons test	Mean Diff.	95.00% CI of diff.	Significant?	Summary	Adjusted P Value
Brain:Male vs. Brain:Female	-1.11	-2.67 to 0.439	No	ns	0.3291
Pituitary:Male vs. Pituitary:Female	-0.0485	-1.60 to 1.50	No	ns	>0.9999
Heart:Male vs. Heart:Female	-1.39	-2.94 to 0.162	No	ns	0.1056
Kidney:Male vs. Kidney:Female	-1.96	-3.52 to -0.412	Yes	**	0.0069
Liver:Male vs. Liver:Female	-0.0116	-1.56 to 1.54	No	ns	>0.9999
Stomach:Male vs. Stomach:Female	-0.579	-2.13 to 0.973	No	ns	0.974
Intestine:Male vs. Intestine:Female	-0.792	-2.34 to 0.760	No	ns	0.7901
Gonad:Male vs. Gonad:Female	0.352	-1.20 to 1.90	No	ns	0.9999
Muscle:Male vs. Muscle:Female	-0.0601	-1.61 to 1.49	No	ns	>0.9999

Table S3: Presents P values for Panel A – Fasting experiment

Tukey's multiple comparisons test	Mean Diff.	95.00% CI of diff.	Significant?	Summary	Adjusted P Value
2:Fed vs. 2:Fasted	-1.1	-2.73 to 0.520	No	ns	0.1614
7:Fed vs. 7:Fasted	1.12	-0.503 to 2.75	No	ns	0.1547
7:Fed vs. 7:Re-fed	-0.172	-1.80 to 1.45	No	ns	0.9932
7:Fasted vs. 7:Re-fed	-1.29	-2.92 to 0.331	No	ns	0.1027

Table S3: Presents P values for Panel B-Fasting experiment

Tukey's multiple comparisons test	Mean Diff.	95.00% CI of diff.	Significant?	Summary	Adjusted P Value
2:Fed vs. 2:Fasted	0.634	-0.795 to 2.06	No	ns	0.4262
7:Fed vs. 7:Fasted	2.79	1.36 to 4.22	Yes	**	0.0045
7:Fed vs. 7:Re-fed	2.51	1.08 to 3.94	Yes	**	0.0067
7:Fasted vs. 7:Re-fed	-0.28	-1.71 to 1.15	No	ns	0.9197

Table S3: Presents P values for Panel C – Fasting experiment

Tukey's multiple comparisons test	Mean Diff.	95.00% CI of diff.	Significant?	Summary	Adjusted P Value
2:Fed vs. 2:Fasted	-0.741	-2.33 to 0.853	No	ns	0.3908
7:Fed vs. 7:Fasted	0.3	-1.29 to 1.89	No	ns	0.9301
7:Fed vs. 7:Re-fed	-0.139	-1.73 to 1.45	No	ns	0.9972
7:Fasted vs. 7:Re-fed	-0.439	-2.03 to 1.15	No	ns	0.7738

Table S3: Presents P values for Panel D-Fasting experiment

Tukey's multiple comparisons test	Mean Diff.	95.00% CI of diff.	Significant?	Summary	Adjusted P Value
2:Fed vs. 2:Fasted	7.62	4.77 to 10.5	Yes	**	0.0013
7:Fed vs. 7:Fasted	3.83	0.974 to 6.69	Yes	*	0.0181
7:Fed vs. 7:Re-fed	3.39	0.536 to 6.25	Yes	*	0.0278
7:Fasted vs. 7:Re-fed	-0.437	-3.29 to 2.42	No	ns	0.9678

Table S3: Presents P values for Panel A-E2 experiment

Tukey's multiple comparisons test	Mean Diff.	95.00% CI of diff.	Significant ?	Summar y	Adjuste d P Value
3h:Control vs. 3h:E2:0.1μM	-0.971	-2.37 to 0.429	No	ns	0.3043
3h:Control vs. 3h:E2:1μM	0.188	-1.21 to 1.59	No	ns	>0.9999
3h:Control vs. 3h:E2:10μM	0.879	-0.522 to 2.28	No	ns	0.4213
6h:Control vs. 6h:E2:0.1μM	-2.89	-4.29 to -1.49	Yes	***	0.0001
6h:Control vs. 6h:E2:1μM	-0.743	-2.14 to 0.658	No	ns	0.6285
6h:Control vs. 6h:E2:10μM	0.0902	-1.31 to 1.49	No	ns	>0.9999
12h:Control vs. 12h:E2:0.1μM	-1.07	-2.47 to 0.330	No	ns	0.2069
12h:Control vs. 12h:E2:1μM	0.554	-0.847 to 1.95	No	ns	0.8912
12h:Control vs. 12h:E2:10μM	0.887	-0.514 to 2.29	No	ns	0.4103

Table S3: Presents P values for Panel B-E2 experiment

Tukey's multiple comparisons test	Mean Diff.	95.00% CI of diff.	Significant?	Summary	Adjusted P Value
3h:Control vs. 3h:E2:0.1μM	0.847	-0.431 to 2.12	No	ns	0.356
3h:Control vs. 3h:E2:1μM	0.298	-0.979 to 1.58	No	ns	0.997
3h:Control vs. 3h:E2:10μM	0.476	-0.802 to 1.75	No	ns	0.921
6h:Control vs. 6h:E2:0.1μM	2.03	-0.753 to 1.31	No	ns	0.0514
6h:Control vs. 6h:E2:1μM	1.54	-0.260 to 2.82	No	ns	0.0539
6h:Control vs. 6h:E2:10μM	1.87	-0.593 to 1.15	No	ns	0.0529
12h:Control vs. 12h:E2:0.1μM	1.06	-0.219 to 2.34	No	ns	0.1431
12h:Control vs. 12h:E2:1μM	0.921	-0.357 to 2.20	No	ns	0.2637
12h:Control vs. 12h:E2:10μM	1.05	-0.226 to 2.33	No	ns	0.1479

Table S3: Presents P values for Panel C - E2 experiment

Tukey's multiple comparisons test	Mean Diff.	95.00% CI of diff.	Significant?	Summary	Adjusted P Value
3h:Control vs. 3h:E2:0.1μM	-3.96	-5.63 to -2.30	Yes	****	<0.0001
3h:Control vs. 3h:E2:1μM	-2.62	-4.28 to -0.958	Yes	**	0.0015
3h:Control vs. 3h:E2:10μM	-2.48	-4.14 to -0.815	Yes	**	0.0025
6h:Control vs. 6h:E2:0.1μM	-0.936	-2.60 to 0.726	No	ns	0.5562

6h:Control vs. 6h:E2:1µM	-0.243	-1.91 to 1.42	No	ns	>0.9999
6h:Control vs. 6h:E2:10µM	0.882	-0.780 to 2.54	No	ns	0.628
12h:Control vs. 12h:E2:0.1µM	0.165	-1.50 to 1.83	No	ns	>0.9999
12h:Control vs. 12h:E2:1µM	0.319	-1.34 to 1.98	No	ns	0.9994
12h:Control vs. 12h:E2:10µM	0.423	-1.24 to 2.09	No	ns	0.994

Table S3: Presents P values for Panel A -MT experiment

Tukey's multiple comparisons test	Mean Diff.	95.00% CI of diff.	Significant?	Summary	Adjusted P Value
3h:Control vs. 3h:MT:0.1µM	-0.521	-1.27 to 0.225	No	ns	0.2968
3h:Control vs. 3h:MT:1µM	-0.416	-1.16 to 0.330	No	ns	0.5675
3h:Control vs. 3h:MT:10µM	0.135	-0.610 to 0.881	No	ns	0.9997
6h:Control vs. 6h:MT:0.1µM	-0.255	-1.00 to 0.491	No	ns	0.9526
6h:Control vs. 6h:MT:1µM	0.221	-0.525 to 0.967	No	ns	0.9815
6h:Control vs. 6h:MT:10µM	0.549	-0.197 to 1.29	No	ns	0.2426
12h:Control vs. 12h:MT:0.1µM	-0.292	-1.04 to 0.454	No	ns	0.8968
12h:Control vs. 12h:MT:1µM	-0.13	-0.876 to 0.616	No	ns	0.9998
12h:Control vs. 12h:MT:10µM	0.219	-0.527 to 0.965	No	ns	0.9824

Table S3: Presents P values for Panel B-MT experiment

Tukey's multiple comparisons test	Mean Diff.	95.00% CI of diff.	Significant?	Summary	Adjusted P Value
3h:Control vs. 3h:MT:0.1µM	-0.698	-1.65 to 0.258	No	ns	0.25
3h:Control vs. 3h:MT:1µM	0.458	-0.498 to 1.41	No	ns	0.7405
3h:Control vs. 3h:MT:10µM	-0.362	-1.32 to 0.593	No	ns	0.9134
6h:Control vs. 6h:MT:0.1µM	-0.976	-1.93 to -0.0204	No	ns	0.0537
6h:Control vs. 6h:MT:1µM	0.317	-0.639 to 1.27	No	ns	0.9606
6h:Control vs. 6h:MT:10µM	-0.635	-1.59 to 0.321	No	ns	0.3528
12h:Control vs. 12h:MT:0.1µM	-1.15	-2.11 to -0.198	No	ns	0.0537
12h:Control vs. 12h:MT:1µM	-0.956	-1.91 to -0.00111	No	ns	0.0596
12h:Control vs. 12h:MT:10µM	-0.827	-1.78 to 0.128	No	ns	0.1143

Table S3: Presents P values for Panel C -MT experiment

Tukey's multiple comparisons test	Mean Diff.	95.00% CI of diff.	Significant?	Summary	Adjusted P Value
3h:Control vs. 3h:MT:0.1µM	-0.621	-1.03 to -0.211	Yes	**	0.0021
3h:Control vs. 3h:MT:1µM	-0.974	-1.38 to -0.564	Yes	****	<0.0001
3h:Control vs. 3h:MT:10µM	-2.05	-2.46 to -1.64	Yes	****	<0.0001
6h:Control vs. 6h:MT:0.1µM	-1.04	-1.46 to -0.635	Yes	****	<0.0001
6h:Control vs. 6h:MT:1µM	-1.36	-1.77 to -0.945	Yes	****	<0.0001
6h:Control vs. 6h:MT:10µM	-1.73	-2.14 to -1.32	Yes	****	<0.0001

12h:Control vs. 12h:MT:0.1μM	-1.02	-1.43 to -0.612	Yes	****	<0.0001
12h:Control vs. 12h:MT:1μM	-1.43	-1.84 to -1.02	Yes	****	<0.0001
12h:Control vs. 12h:MT:10μM	-2	-2.41 to -1.59	Yes	****	<0.0001
